# A High-Throughput Antibody-Based Microarray Typing Platform

**DOI:** 10.3390/s130505737

**Published:** 2013-05-03

**Authors:** Gehring Andrew, Barnett Charles, Ted Chu, Chitrita DebRoy, Doris D'Souza, Shannon Eaker, Pina Fratamico, Barbara Gillespie, Narasimha Hegde, Kevin Jones, Jun Lin, Stephen Oliver, George Paoli, Ashan Perera, Joseph Uknalis

**Affiliations:** 1 Molecular Characterization of Foodborne Pathogens Research Unit, United States Department of Agriculture-North Atlantic Area-Agricultural Research Service-Eastern Regional Research Center, Wyndmoor, PA 19038, USA; E-Mails: ted.chu@ars.usda.gov (T.C.); pina.fratamico@ars.usda.gov (P.F.); george.paoli@ars.usda.gov (G.P.); joseph.uknalis@ars.usda.gov (J.U.); 2 NanoDetection Technology, Inc., Franklin, OH 45005, USA; E-Mails: Charlie@ndtbio.com (C.B.); shannoneaker@yahoo.com (S.E.); drkevinjones@comcast.net (K.J.); bobperera@gmail.com (A.P.); 3 * E. coli* Reference Center, Department of Veterinary and Biomedical Sciences, Pennsylvania State University, University Park, PA 16802, USA; E-Mails: rcd3@psu.edu (C.D.); nvh1@psu.edu (N.H.); 4 Department of Food Science and Technology, University of Tennessee, Knoxville, TN 37916, USA; E-Mail: ddsouza@utk.edu; 5 Department of Animal Science, University of Tennessee, Knoxville, TN 37916, USA; E-Mails: bgillesp@utk.edu (B.G.); jlin6@utk.edu (J.L.); soliver@utk.edu (S.O.)

**Keywords:** antibody, microarray, bacteria, fluorescence, microtiter plate, typing

## Abstract

Many rapid methods have been developed for screening foods for the presence of pathogenic microorganisms. Rapid methods that have the additional ability to identify microorganisms via multiplexed immunological recognition have the potential for classification or typing of microbial contaminants thus facilitating epidemiological investigations that aim to identify outbreaks and trace back the contamination to its source. This manuscript introduces a novel, high throughput typing platform that employs microarrayed multiwell plate substrates and laser-induced fluorescence of the nucleic acid intercalating dye/stain SYBR Gold for detection of antibody-captured bacteria. The aim of this study was to use this platform for comparison of different sets of antibodies raised against the same pathogens as well as demonstrate its potential effectiveness for serotyping. To that end, two sets of antibodies raised against each of the “Big Six” non-O157 Shiga toxin-producing *E. coli* (STEC) as well as *E. coli* O157:H7 were array-printed into microtiter plates, and serial dilutions of the bacteria were added and subsequently detected. Though antibody specificity was not sufficient for the development of an STEC serotyping method, the STEC antibody sets performed reasonably well exhibiting that specificity increased at lower capture antibody concentrations or, conversely, at lower bacterial target concentrations. The favorable results indicated that with sufficiently selective and ideally concentrated sets of biorecognition elements (e.g., antibodies or aptamers), this high-throughput platform can be used to rapidly type microbial isolates derived from food samples within *ca.* 80 min of total assay time. It can also potentially be used to detect the pathogens from food enrichments and at least serve as a platform for testing antibodies.

## 1. Introduction

The U.S. Centers for Disease Control and Prevention estimates that 31 major foodborne pathogens account for approximately 9.4 million illnesses; 56,000 hospitalizations; and 1,350 deaths per year in the United States alone [1]. Microbial culture methods are the “gold standard” for detection and identification of pathogenic bacteria in foods. These methods combine growth enrichment, plating onto selective and/or differential agars, as well as biochemical tests for confirmatory analysis. Though powerful enough to detect a single, specific bacterium, they may require days or weeks to complete and typically do not produce quantitative data. Rapid detection of a few, targeted bacteria in complex food matrices, requires methods of extraordinary sensitivity and specificity. Such detection techniques are termed “rapid methods” and they are frequently employed for the screening of foods in order to detect the presence of potentially pathogenic microorganisms [2–5]. In addition to detection, there also exists multiple means for the relatively rapid classifying/categorizing or “typing” bacteria using phenotyping and genotyping strategies [6]. Some of these methods are cumbersome and labor-intensive especially if numerous subtypes exist for a given species. Therefore, faster and simpler typing alternatives are ideally required as tools for rapid epidemiological investigations.

Detection microarrays, employing biorecognition elements that include nucleic acid probes or antibodies, have been proven to be advantageous as rapid methods for the high-throughput, multiplexed detection of foodborne bacterial pathogens and toxins [7–9]. In this study, the high capacity of microarray to interrogate samples with numerous biorecognition elements was harnessed using a quick, universal labeling technique. The assay was demonstrated using the Shiga-toxin producing *E. coli* (STEC), *E. coli* O157:H7 as well as the “Big Six” non-O157 STEC, captured by antibodies and detected via labeling with a fluorescent, DNA intercalating stain. Though similar to a notable single tube-based microarray O-antigen typing assay for *E. coli* that employed a universal anti-LPS core antibody labeling approach [10], this typing microarray was conducted in individual wells of 96-well plates and could be used to rapidly screen and type large numbers of food samples for pathogens in a high-throughput manner.

## 2. Experimental Section

### 2.1. Materials

Reagents used in this research were: phosphate-buffered saline (PBS; 10 mM phosphate, 2.7 mM KCl, 137 mM NaCl, pH 7.4) tablets, glycerol, Tween 20, Tris-buffered saline (TBS; 10 mM Tris-HCl, 50 mM NaCl, pH 8.0), and bovine serum albumin (BSA; fraction V) from Sigma (St. Louis, MO, USA). Plates used were MicroAmp^®^ 384-well reaction plates (polypropylene, conical wells) from PE Biosystems (Carlsbad, CA, USA) which served as microarray “source” plates and antibodies were printed into black-walled, clear/transparent and flat-bottomed, polystyrene 96-multiwell microtiter plates with high binding (FLUOTRAC 600) surfaces from Greiner Bio-One North America Inc. (Monroe, NC, USA) which served as “destination” plates. Antibodies to *E. coli* were obtained from Kirkegaard & Perry Laboratories, Inc. (affinity purified IgGs; KPL; Gaithersburg, MD, USA) and the Pennsylvania State University *E. coli* Reference Center (protein A purified IgGs; University Park, PA, USA). Anti-Shiga toxin-1 (Stx-1) antibody (from Toxin Technology, Sarasota, FL, USA) was labeled with Alexa Fluor 555 (from Invitrogen, Carlsbad, CA, USA) according to kit instructions and used as a microarray fluorescent marker. *E. coli* O157:H7 strain B1409 was from Centers for Disease Control and Prevention (Atlanta, GA, USA), other bacterial strains were obtained from in-house stocks. Luria-Bertani broth was from Becton Dickinson (Sparks, MD, USA). SYBR Gold was obtained from Invitrogen. Any chemicals not mentioned were at least of reagent grade.

### 2.2. Apparatus

Antibody solutions were printed into 96-well microplate wells using a Gene Machine Omnigrid Accent from Bucher (Basel, Switzerland) that held a single, SMP3 printing pin (TeleChem International, Inc., Sunnyvale, CA, USA). Fluorescent scans of the microarrayed-microtiter plates were acquired with an LS400 laser scanner from Tecan (Research Triangle Park, NC, USA). Centrifugation of microtiter plates was conducted in an Eppendorf model 5810R refrigerated centrifuge outfitted with an A-4-62 swinging bucket rotor (Eppendorf AG, Hamburg, Germany). UV-Vis spectrophotometric measurements were made with a Cary 50 UV-Vis scanning spectrophotometer (Varian, Inc., Palo Alto, CA, USA). A Petroff-Hausser counting chamber from Thomas Scientific (Swedesboro, NJ, USA) was used to enumerate bacterial cells.

### 2.3. Growth and Enumeration of Bacteria

Individual colonies of bacteria were inoculated into 25 mL of modified Luria-Bertani broth. This was incubated at 37 °C for 18 h with shaking at 160 rpm. Serial dilutions of cultures were enumerated in quadruplicate with a Petroff-Hausser counting chamber as described by Gehring, *et al.* [11].

### 2.4. Antibody Preparation and Microarray Printing

The non-biotinylated anti-*E. coli* capture antibodies were reconstituted in 50% glycerol to 1 mg/mL and diluted to various concentrations in PBS containing 5% glycerol for array printing. (The relatively high concentration of glycerol was maintained in order to prevent evaporation of the droplets and maintain a hydrated state for the capture antibodies [12].)

Approximately 25 μL of thoroughly-mixed capture antibody solution was pipetted into separate wells of MicroAmp source plates on the microarray printer (located on a thermal block maintained at 4 °C). In order to remove any air bubbles, the plates were centrifuged at 1,000 rpm (200 × g) for 2 min immediately prior to printing. Array printing was performed using the following settings: preprints/blots = 20; contact time = 0; dip and print velocity = 2 cm/s; dip and print acceleration = 10 cm/s^2^, with an SMP3 (100 μm spot diameter) pin, which delivered a volume of approx. 0.7 nL per contact stroke. The pins were manually sonicated for 5 min in distilled H_2_O after each daily printing routine. Columns of 8 spots per each antibody were printed with a spot separation, from edge-to-edge, of 150 μm in both “X-axis” and “Y-axis” directions. After printing, all wells were visually examined to ensure that spots were uniformly printed. Upon completion of printing, the spotted destination plates sat at RT for 1 h prior to use.

### 2.5. Antibody Microarray Detection of Bacteria in Multiwell Plates

**Figure 1.**
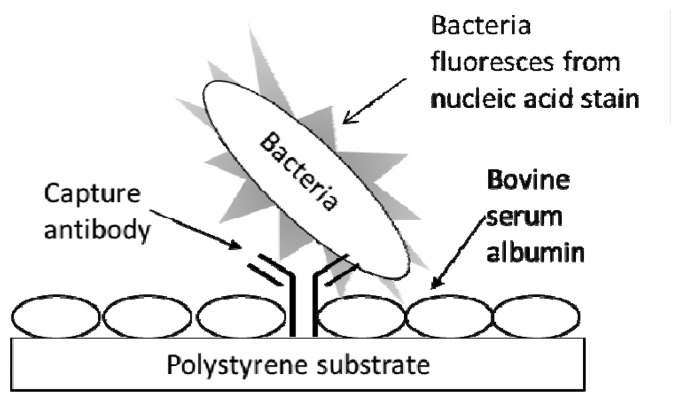
Schematic for the antibody-based typing microarray. Black-walled, clear-bottomed polystyrene 96-well microtiter plates were array-printed with capture antibodies, the remainder of the polystyrene surface was blocked with BSA, and bacterial sample was introduced and conferred fluorescent with a nucleic acid stain. Laser-induced fluorescence was used to detect captured bacteria.

The procedure for conducting a fluorescent immunoassay (Figure 1) in the multiwell antibody microarray detection of bacteria generally followed the one previously described for microarray slides [13] with several modifications. All immunoassay procedures and reagents were at RT. Wells of the destination plate, preprinted with capture antibody, were washed by being filled with 200 μL PBST (PBS containing 0.05% Tween 20), immediately emptied by rapidly inverting the plate, and residual liquid was removed by striking the upside down plate onto a paper towel on the lab bench. This wash procedure was repeated once with PBST. The plate wells were blocked with 200 μL of 1% BSA in PBS for 30 min. The BSA solution was removed and the plate was washed as above. Analyte (100 μL) was then added, and each plate was subjected to centrifugation for 5 min at 4,000 RPM (*ca.* 3200 RCF) to promote analyte capture. The wells were washed twice with PBST and excess liquid was removed as above. Next, 50 μL SYBR Gold reporter solution was added to each well that was subjected to static incubation for 1 h at RT. Wells were washed twice with PBST, excess liquid was removed, the bottom of the plate was wiped clean with an ethanol-soaked tissue, the bottom was spray-dried with canned air, and then the plate was inverted and scanned (fluorescence acquisition parameters—excitation: 543 nm, emission filter: 590 nm) on the array scanner using single channel mode. During reporter incubation and prior to measurement, LS400 scanner lasers were turned on to warm up and stabilize for 30 min. Typical LS400 instrument scanning parameters, set and controlled via the Array-Pro Analyzer software (ver. 4.5.1.73) interface included: autofocusing in well mode, PMT gain that ranged from 100–150, 20 μm resolution, small pinhole setting, and optimization of integration time = 1.

## 3. Results and Discussion

Initial tests were conducted with Pennsylvania State University (PSU) antisera raised against each of the Big Six STECs. However, none of the bacteria tested were detected (*i.e.*, captured) by the printed antisera (data not shown). A plausible explanation for this observation was that the antisera contained numerous globular proteins, including non-specific immunoglobulins, that either blocked the binding of anti-STEC antibodies or masked their effectiveness. Therefore, subsequent testing was conducted with immunoglobulin G (IgG) purified from the antisera using Protein A [14].

Since this work focuses on proof-of-concept for a novel typing array, the data was compiled and presented in a semi-quantitative manner. This was not only justified by qualitative observation being sufficient for determining positive *versus* negative responses (from visual analysis of induced-fluorescence scanned images), but also based upon observations that purified PSU IgGs, diluted over 3 orders of magnitude in concentration, did not elicit any discernible difference in response at constant levels of bacterial cell exposure.

Figure 1 exhibits the schematic for the typing microarray assay. The assay involved array printing of IgGs at two levels of concentration (1:10 to 1:30 at low *versus* 1:100 to 1:1,000 at high dilution levels) and virtually instantaneous adsorption of the antibodies onto inexpensive polystyrene substrates (*i.e.*, the clear bottom of black-walled, microtiter 96-multiwell plates). Unbound sites were blocked with BSA to prevent non-specific adsorption of bacterial cells. Serially diluted samples of live bacterial cells were added to the wells and the entire multiwell plate was centrifuged for 5 min to ensure contact of the cells to the capture antibodies. After washing, the cells were labeled with the nucleic acid stain, SYBR Gold, the wells were washed again, and the plate was subjected to laser-induced fluorescence in a microarray scanner.

**Figure 2.**
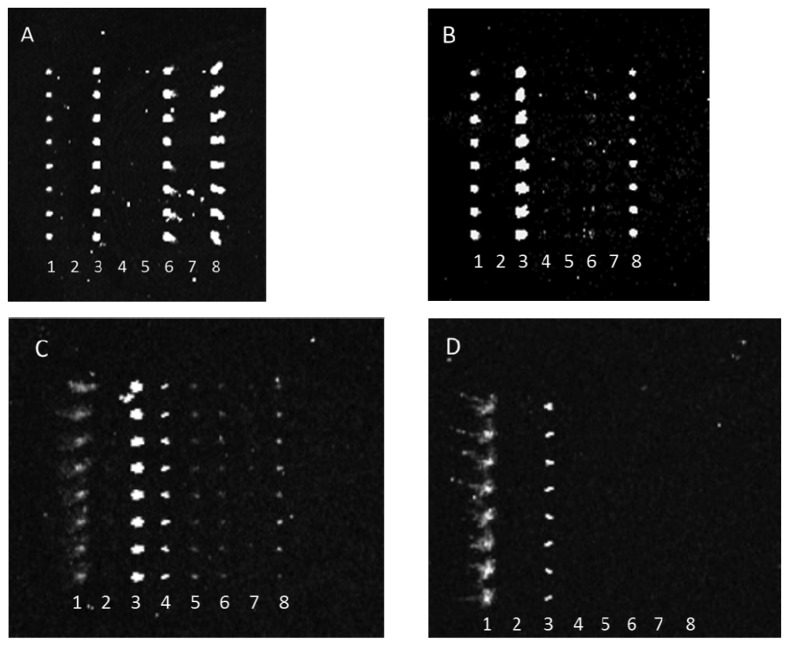
Laser-induced fluorescence scan images of anti-STEC microarrays: Dilution of capture antibody increased specificity to bacterial target in typing microarray. Multiple anti-bacterial capture antibodies were array-printed onto the bottoms of single wells of 96-well microtiter plates. Each column (or lane) within the array was printed with the same antibody, providing 8 replicates. The array was printed as follows: column 1, AF555-labeled antibody marker; columns 2-8, anti-O157, anti-O145, anti-O126, anti-O111, anti-O103, anti-O45, and anti-O26 antibodies, respectively. Samples containing purified bacteria were added to separate, individual wells. After any binding of the bacteria to capture antibody, captured bacteria were visualized via exposure to SYBR Gold and detection with laser-induced fluorescence scanning. Each image, A-D, depicts a single array and reveals the reaction of *ca.* 1 × 10^8^ CFU/mL of: *E. coli* O26 with PSU anti-STEC IgGs at low (1:10; A) and high (1:500; B) dilutions, and *E. coli* O145 reacted with KPL anti-STEC IgGs at low (1:30; C) and high (1:100; D) dilutions.

Antibody-mediated capture of bacterial cells was observed in scanned images that revealed spots of fluorescence. Typical examples of these images are represented in Figure 2. Figure 2 reveals that dilution of either the PSU or Kirkegaard & Perry Laboratories, Inc. (KPL) IgG sets appeared to reduce cross-reactivity for the same bacterial target, hence indicating that dilution of capture antibodies resulted in improved specificity. This observation can be explained by realization that apparently lower concentrations of poorly binding antibodies (*i.e.*, those with lower dissociation constants) are outcompeted by more tightly binding, specific IgGs. Note, these images were not normalized, so though fluorescence intensity appeared to increase from Figure 2(A,B) suggesting higher binding of *E. coli* O26 to anti-O145 antibody, quantitative analysis revealed that there was no significant change in net response unlike the significant decrease in reaction for that serotype with anti-O103. Therefore, the results indicate a higher affinity to anti-O145 than anti-O103 for *E. coli* O26.

**Figure 3.**
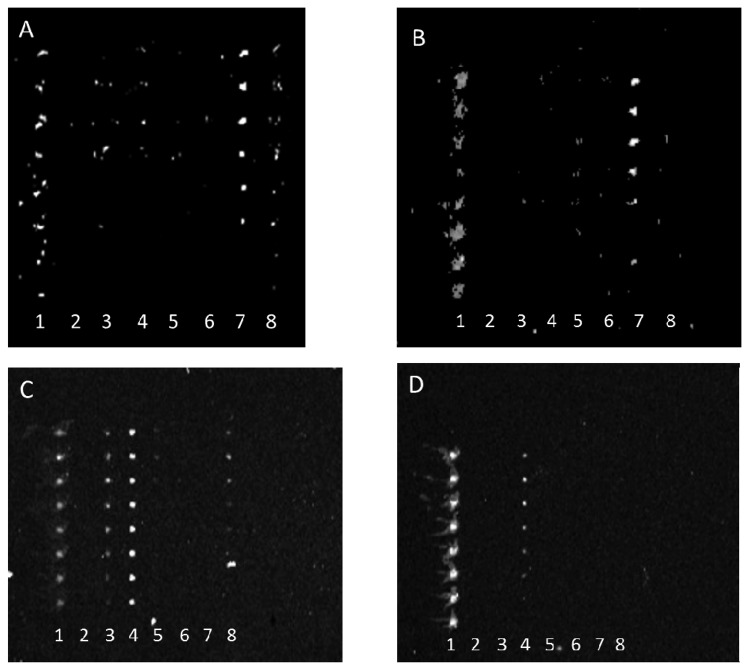
Laser-induced fluorescence scan images of anti-STEC microarrays: Dilution of bacterial target increased specificity of antibody capture in typing microarray. Multiple anti-bacterial capture antibodies were array-printed onto the bottoms of single wells of 96-well microtiter plates. Each column (or lane) within the array was printed with the same antibody, providing 8 replicates. The array was printed as follows: column 1, AF555-labeled antibody marker; columns 2-8, anti-O157, anti-O145, anti-O126, anti-O111, anti-O103, anti-O45, and anti-O26 antibodies, respectively. Samples containing purified bacteria were added to separate, individual wells. After any binding of the bacteria to capture antibody, captured bacteria were visualized via exposure to SYBR Gold and detection with laser-induced fluorescence scanning. Each image, A-D, depicts a single array and reveals the reaction of low dilutions of anti-STEC IgGs with different concentrations of STEC cells: PSU IgGs (diluted 1:10) with *E. coli* O45 at concentrations of *ca.* 1 × 10^8^ CFU/mL (**A**) and *ca.* 1 × 10^7^ CFU/mL (**B**) and KPL IgGs (diluted 1:30) reacted with *E. coli* O121 at concentrations of *ca.* 1 × 10^8^ CFU/mL (**C**) and *ca.* 1 × 10^7^ CFU/mL (**D**).

Conversely, antibody array specificity also improved when bacterial target was relatively diluted as exhibited in Figure 3.

This observation similarly indicates that either the reaction of weakly binding antibodies was rate limited with decreasing bacterial concentration or that the antibody fractions with the greatest specificity were at the highest concentrations in each anti-STEC IgG pool and therefore their binding was only observed at lower concentrations of bacterial target. Note, because of poor printing of capture antibody, the aberrant spots in Figure 3(A) were not artifacts, but actual positive signals that indicated cross-reactivity of multiple capture antibodies with *E. coli* O45. For example, the absence of spots in column 8 of Figure 3(B), relative to column 8 of Figure 3(A), clearly shows diminishment of such cross-reactive signals.

Tables 1, 2, 3 and 4 reveal semi-quantitative responses for target, as well as non-target bacteria with the respective sets of KPL and PSU IgG antibodies at either low or high dilution levels. As representative in Figure 2, higher dilutions of the antibodies typically conferred greater specificity of interaction. Of particular interest, the typing array also reveals relative titer levels as indicative of strong *versus* weak fluorescence response. Select, closely related bacteria (e.g., *Shigella spp.* and *Citrobacter spp.*, which along with *E. coli*, are all members of the family Enterobacteriaceae) were demonstrated to elicit false positive responses for both sets of antibodies. Most unexpected was that one strain of *E. coli* K-12 (ATCC 24425) heavily cross-reacted with both antibody sets. K-12 strains are known as “non-decorated” or “rough” strains that lack O-antigenic polysaccharide side chains in their lipopolysaccharide (LPS) coatings. (The LPS of these rough mutants is made up of only the Lipid A molecule and core oligosaccharides.) It may be that all anti-*E. coli* antibody sets contained antibodies that bind to the LPS core and that the core is accessible to the anti-core antibodies in the rough mutants. However, the (Shigatoxigenic) *E. coli* (used in this study) contained “decorated” (*i.e.*, O-antigen polysaccharide modified) LPS that may sterically hinder and thus mask the binding of the anti-core antibodies to the LPS core. Additional non-pathogenic *E. coli* strains were tested to determine if the cross-reactivity was due to binding of the anti-STEC polyclonal IgGs to the K-12 LPS core.

The *E. coli* Crooks strain, another rough strain that contains a different LPS core structure (R1 core) [15], and two additional strains derived from strain K-12 (JM107 and TG1) did not exhibit the same cross-reactivity observed for K-12 strain ATCC 29425. The absence of cross-reactivity with these three strains suggests that the observed cross-reaction with *E. coli* K-12 is not due to binding anti-STEC IgGs to either the K-12 type or R1 core, but rather to some epitope unique to the ATCC 29425 strain of K-12. The nature of the *E. coli* K-12 cross-reacting epitope(s) remains to be determined.

## 4. Conclusions/Outlook

A multiwell, multiplex antibody microarray platform was developed that may find application in the detection and typing of bacterial cells, as well as antibody testing. The array platform was demonstrated with the simultaneous typing of *E. coli* O157:H7 and Big Six STECs in a high-throughput, inexpensive, and rapid (*ca.* 80 min) assay. With improved biorecognition elements (antibodies, nucleic acids, aptamers, *etc.*) this procedure may be used to rapidly screen and type large numbers of food samples for STEC and additional pathogenic bacteria.

## Figures and Tables

**Table 1.** Semi-quantitative microarray responses for target and non-target bacterial samples (*ca.* 1 × 10^8^ CFU/mL) reacted with low dilutions (1:10 to 1:30) of KPL purified IgGs. (Key: − = no binding; (+) = faint binding; + = binding; ++ = high binding, nd = not determined).

**Target**	**Capture Antibody**						
	O26	O45	O103	O111	O121	O145	O157
*Escherichia coli*							
O26:H11 (H19)	++	−	−	−	−	−	−
O45 (B8026-C1)	+	++	−	−	−	−	−
O103 (DA-33)	−	−	+	−	−	−	−
O111:NM (3007-85)	+	+	−	++	−	−	−
O121 (DA-1)	−	−	−	−	−	−	−
O145:NM (SJ23)	−	+	−	−	−	++	−
O157:H7 (B1409)	−	−	−	−	−	−	++
O157:H7 (SEA 13B88)	nd	nd	nd	nd	nd	nd	nd
K-12 (ATCC 29425)	+	++	+	+	+	+	+
TG1 (K-12 derivative)	−	−	−	−	−	−	−
Crooks (ATCC 8739)	−	−	−	−	−	−	−
JM107 (K-12 derivative)	−	−	−	−	−	−	−
*Salmonella* Virchow	nd	nd	nd	nd	nd	nd	nd
*Citrobacter freundii* (ATCC 8090)	nd	nd	nd	nd	nd	nd	nd
*Shigella sonnei* (A11)	+	+	(+)	(+)	(+)	(+)	(+)

**Table 2.** Semi-quantitative microarray responses for target and non-target bacterial samples (*ca.* 1 × 10^8^ CFU/mL) reacted with low dilutions (1:10 to 1:30) of PSU purified IgGs. (Key: − = no binding; (+) = faint binding; + = binding; ++ = high binding, nd = not determined).

**Target**	**Capture Antibody**						
	O26	O45	O103	O111	O121	O145	O157
*Escherichia coli*							
O26:H11 (H19)	++	(+)	+	(+)	(+)	++	+
O45 (B8026-C1)	(+)	+	−	(+)	(+)	(+)	(+)
O103 (DA-33)	(+)	−	+	−	−	(+)	(+)
O111:NM (3007-85)	−	−	−	+	−	−	−
O121 (DA-1)	(+)	−	(+)	−	+	−	(+)
O145:NM (SJ23)	(+)	−	−	−	−	++	(+)
O157:H7 (B1409)	−	−	−	−	−	−	++
O157:H7 (SEA 13B88)	nd	nd	nd	nd	nd	nd	nd
K-12 (ATCC 29425)	++	+	++	+	++	++	+
TG1 (K-12 derivative)	−	−	−	−	−	−	−
Crooks (ATCC 8739)	−	−	−	−	−	−	−
JM107 (K-12 derivative)	−	−	−	−	−	−	−
*Salmonella* Virchow	−	−	−	−	−	−	−
*Citrobacter freundii* (ATCC 8090)	−	−	+	(+)	−	−	−
*Shigella sonnei* (A11)	+	+	(+)	(+)	(+)	(+)	+

**Table 3.** Semi-quantitative microarray responses for target and non-target bacterial samples (*ca.* 1 × 10^8^ CFU/mL) reacted with high dilutions (1:100 to 1:500) of KPL purified IgGs. (Key: − = no binding; (+) = faint binding; + = binding; ++ = high binding, nd = not determined).

**Target**	**Capture Antibody**						
	O26	O45	O103	O111	O121	O145	O157
*Escherichia coli*							
O26:H11 (H19)	++	−	−	−	−	−	−
O45 (B8026-C1)	(+)	(+)	−	(+)	−	−	−
O103 (DA-33)	−	−	(+)	−	−	−	−
O111:NM (3007-85)	−	−	−	+	−	−	−
O121 (DA-1)	−	−	−	−	(+)	−	−
O145:NM (SJ23)	(+)	−	−	(+)	−	+	−
O157:H7 (B1409)	−	−	−	−	−	−	+
O157:H7 (SEA 13B88)	−	−	−	−	−	−	+
K-12 (ATCC 29425)	++	+	+	+	(+)	(+)	(+)
TG1 (K-12 derivative)	−	−	−	−	−	−	−
Crooks (ATCC 8739)	−	−	−	−	−	−	−
JM107 (K-12 derivative)	−	−	−	−	−	−	−
*Salmonella* Virchow	nd	nd	nd	nd	nd	nd	nd
*Citrobacter freundii* (ATCC 8090)	nd	nd	nd	nd	nd	nd	nd
*Shigella sonnei* (A11)	nd	nd	nd	nd	nd	nd	nd

**Table 4.** Semi-quantitative microarray responses for target and non-target bacterial samples (*ca.* 1 × 10^8^ CFU/mL) reacted with high dilutions (1:500 to 1:1000) of PSU purified IgGs. (Key: − = no binding; (+) = faint binding; + = binding; ++ = high binding, nd = not determined).

**Target**	**Capture Antibody**						
	O26	O45	O103	O111	O121	O145	O157
*Escherichia coli*							
O26:H11 (H19)	++	−	−	−	−	+	−
O45 (B8026-C1)	(+)	++	(+)	−	−	−	−
O103 (DA-33)	-	(+)	++	−	−	−	−
O111:NM (3007-85)	−	−	−	+	−	−	−
O121 (DA-1)	nd	nd	nd	nd	nd	nd	nd
O145:NM (SJ23)	−	−	−	−	−	++	−
O157:H7 (B1409)	−	−	−	−	−	−	++
O157:H7 (SEA 13B88)	−	−	−	−	−	−	nd
K-12 (ATCC 29425)	+	+	++	(+)	++	(+)	−
TG1 (K-12 derivative)	nd	nd	nd	nd	nd	nd	nd
Crooks (ATCC 8739)	nd	nd	nd	nd	nd	nd	nd
JM107 (K-12 derivative)	nd	nd	nd	nd	nd	nd	nd
*Salmonella* Virchow	nd	nd	nd	nd	nd	nd	nd
*Citrobacter freundii* (ATCC 8090)	nd	nd	nd	nd	nd	nd	nd
*Shigella sonnei* (A11)	nd	nd	nd	nd	nd	nd	nd
